# The Network Initiative for Conservation Science (NICS): a model of collaboration and resource sharing among neighbor museums

**DOI:** 10.1186/s40494-021-00559-4

**Published:** 2021-07-28

**Authors:** Federica Pozzi, Elena Basso

**Affiliations:** 1 Department of Scientific Research, The Metropolitan Museum of Art, 1000 Fifth Avenue, New York, NY 10028 USA; 2 Present Address: Center for Conservation and Restoration of Cultural Heritage “La Venaria Reale”, Via XX Settembre 18, 10078 Venaria Reale, Torino Italy

**Fig. 1:**
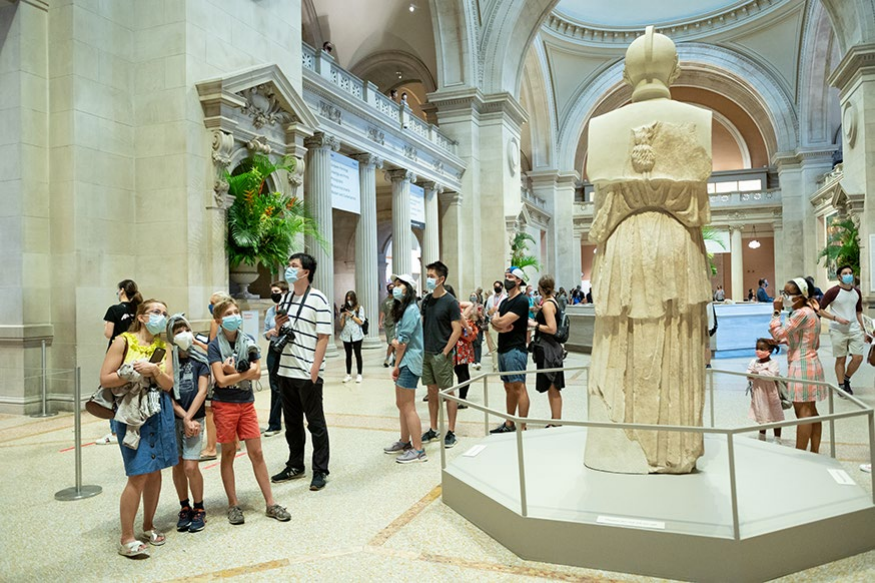
The Met’s Great Hall hosts visitors upon reopening on August 29th, 2020, following a prolonged closure due to the Covid-19 crisis (© The Metropolitan Museum of Art. Photo by Paula Lobo)

Each year, millions of visitors crowd the halls of the vast ensemble of museums in New York City, among which are some of the most prestigious and highly visited cultural heritage sites in the United States (Fig. 1). These halls are home to uncountable objects of untold monetary, cultural, and historic value, many dating back thousands of years, and each demanding specific care to be passed on to future generations. While falling primarily to art conservators, the preservation of these artifacts also relies on the emerging field of conservation science—a technically complex field that lies at the intersection of chemistry, physics, biology, geology, materials science, and engineering. The scientific study of objects of artistic, archaeological, and historical significance often constitutes a multifaceted endeavor, combining compositional analysis, dating, degradation studies, and environmental monitoring. Among the particular challenges typically encountered in this field are, for instance, the need for minimally invasive tests and, sometimes, the impossibility to safely move the artworks outside of museum galleries, storages, or conservation studios for analysis. Moreover, different materials have different requirements in terms of temperature, humidity, and exposure to light, thus necessitating tailored care. While expressing a similar interest in conducting research on their own art collections, New York City museums have also shared many of the same concerns when trying to maintain optimal conservation conditions.

The Metropolitan Museum of Art (The Met) and The Museum of Modern Art (MoMA) are currently the only two New York City museums with full-time scientific staff. In addition to carrying out research in house, scientists at both institutions have actively pursued successful collaborations with colleagues in academia. While conservators may have varying degrees of scientific training and access to basic instrumentation, a scientist’s highly specialized skills and broad technical expertise are often desirable to embark on in-depth investigations and address high-impact questions. To meet the scientific needs of the New York City museum community and nurture opportunities for collaboration among neighbor institutions in the cultural heritage field, in September 2016 The Met’s Department of Scientific Research (DSR) launched the Network Initiative for Conservation Science, or NICS, with financial support from The Andrew W. Mellon Foundation. Growing out of a history of informal collaborations between The Met’s DSR and other New York City museums, NICS is a 6-year program aiming to share The Met’s resources, expertise, and state-of-the-art scientific research facilities with partner institutions free of charge. NICS currently supports 11 museums and cultural heritage institutions in New York City, including the American Museum of Natural History, the Brooklyn Museum, the Central Park Conservancy, the Cooper-Hewitt Smithsonian Design Museum, The Frick Collection, the Hispanic Museum & Library, The Morgan Library & Museum, MoMA, The New York Public Library, the Solomon R. Guggenheim Museum, and The Whitney Museum of American Art.

**Fig. 2:**
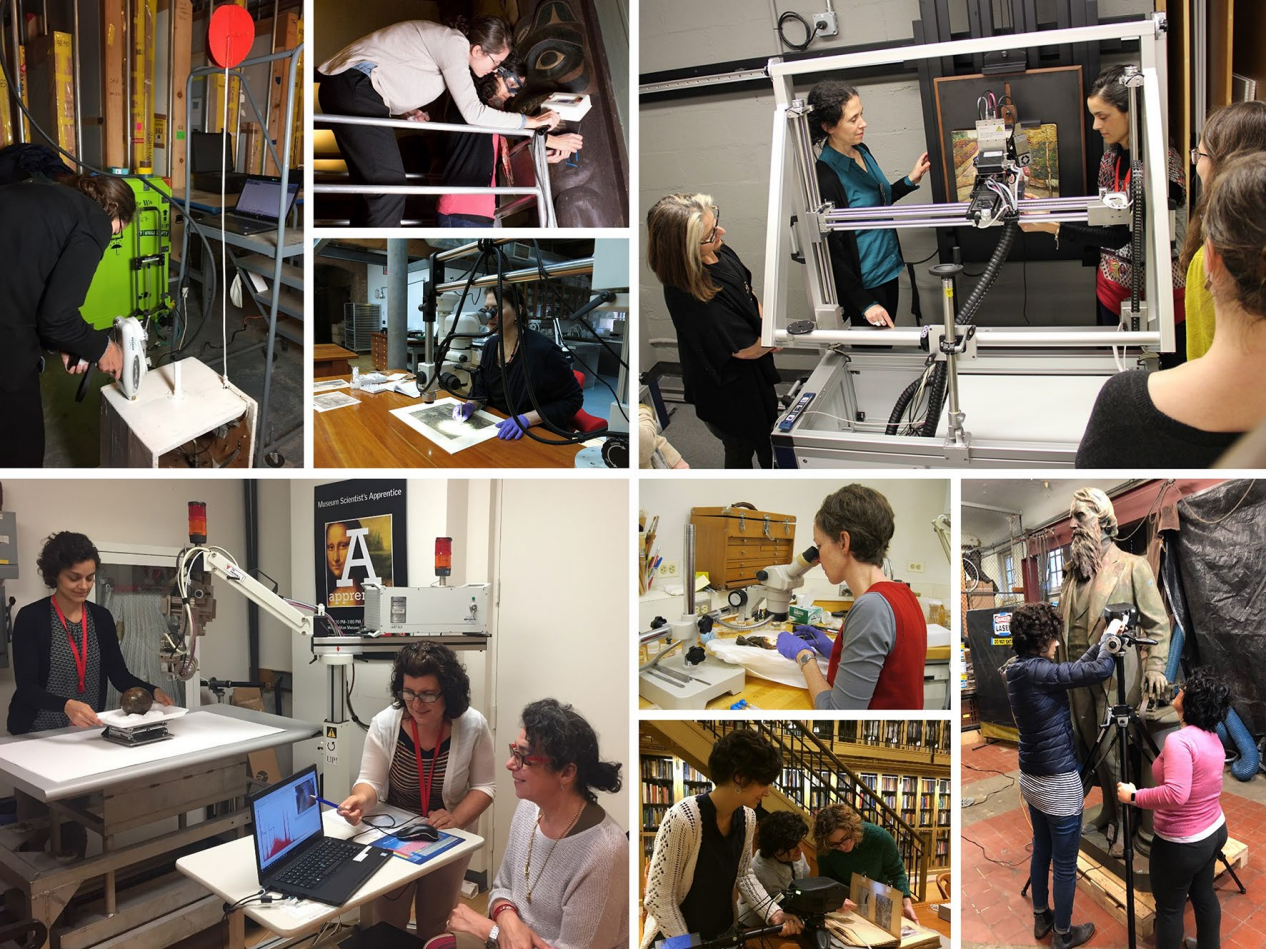
The NICS team examines objects in collaboration with other Met scientists and conservators from partner institutions in New York City

**Fig. 3:**
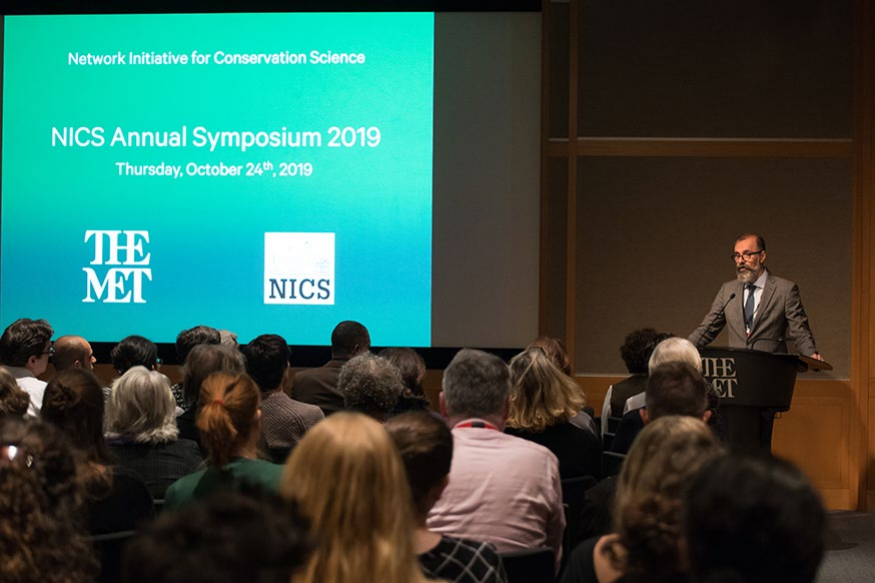
Dr. Marco Leona, The Met’s David H. Koch Scientist in Charge, speaks at the third NICS Annual Symposium, held at the museum on Thursday, October 24th, 2019 (© The Metropolitan Museum of Art. Photo by Paula Lobo)

NICS aims to promote sustainable research and advance scholarship in art history, archaeology, conservation, and science; to provide in-depth characterization of the materials and techniques used to create artifacts of archaeological, historical, and artistic relevance; to offer insight into the state of preservation and degradation of artworks; to develop innovative methodologies for the monitoring, stabilization, and repair of these objects; and to create an interface through which needs, capabilities, and knowledge can be shared at no cost among all institutions in the program. To date, NICS has served as a crucial resource for New York City museums and cultural heritage institutions, allowing members to probe in-depth scientific research questions as well as answer basic requests in the service of art conservation and preservation. Along with the use of benchtop instrumentation available in The Met’s DSR, NICS scientists have created a mobile laboratory that can be employed for in-situ analysis of unmovable objects, and have also relied on external research facilities through national and international collaborations. Since the beginning of the program, the team has carried out collaborative work on 70 projects, focusing on over 300 objects that span a broad range of media, cultures, and historical periods (Fig. 2). Presently at the end of its fifth year, NICS has hosted three Annual Symposia that provided an interdisciplinary forum for over 130 scientists, conservators, and curators, to showcase collaborative work as well as share experiences and ideas in all areas of cultural heritage research (Fig. 3).

This collection, including 8 articles, presents findings from some of the most relevant and fascinating projects that the NICS team has pursued in collaboration with other New York City museums. Among the topics discussed are discoveries on the repainting of Alexander Calder’s motorized sculptures [1], the degradation and cleaning of Central Park outdoor bronze statues [2], the secret drawing practices of Thomas Gainsborough [3], the indigenous decorative technique of a 17th-century lacquered gourd from Columbia [4], the manufacture of Italian Renaissance statuettes by Bertoldo di Giovanni [5], the fleeting colors and surface alterations of Van Gogh paintings [6], the materials of historical photographs from Arctic expeditions [7], and museum-applied surface coatings and the original polychromy of Tsimshian house posts [8]. We hope that the continued activity of NICS in support of scientific research at New York City museums may serve as a networking model for inter-museum collaboration and resource sharing, contributing to the growth of the cultural heritage field and its burgeoning community.
